# To Wnt or Lose: The Missing Non-Coding Linc in Colorectal Cancer

**DOI:** 10.3390/ijms18092003

**Published:** 2017-09-20

**Authors:** Peng Shen, Martin Pichler, Meng Chen, George A. Calin, Hui Ling

**Affiliations:** 1 Department of Experimental Therapeutics, The University of Texas MD Anderson Cancer Center, Houston, TX 77030, USA; shenbo20110311@163.com (P.S.); martin.pichler@medunigraz.at (M.P.); MChen11@mdanderson.org (M.C.); 2 Nanfang Hospital, Southern Medical University/The First School of Clinical Medicine, Southern Medical University, Guangzhou 510515, Guangdong, China; 3 Research Unit of Non-Coding RNA and Genome Editing in Cancer, Division of Oncology, Medical University of Graz, Graz 8010, Austria; 4 The Center for RNA Interference and Non-Coding RNAs, The University of Texas MD Anderson Cancer Center, Houston, TX 77030, USA

**Keywords:** Wnt, long non-coding RNA, CCAT1, CCAT2, PVT1, H19

## Abstract

Colorectal cancer (CRC) is the third most frequent cancer and one of the leading causes for cancer-related mortality. Aberrant activation of the Wnt signaling is an essential initiating factor in colon carcinogenesis, and a driving force of CRC progression. Recently, long non-coding RNAs (lncRNAs) have emerged as significant players in CRC pathogenesis through diversified mechanisms. Although both Wnt signaling and lncRNAs represent interesting research areas for CRC, an effort of directly connecting these two areas is lacking. To fill in the knowledge gap, we focus on the reported findings of lncRNAs that regulate Wnt signaling or essential Wnt signaling targets. These include several newly discovered lncRNAs originated from the amplified cancer-associated chromosome 8q24 region that surrounds the essential Wnt target *MYC* gene, lncRNAs reported to be involved in CRC stem cells, and several individual lncRNAs connected to Wnt signaling through other mechanisms. This review will provide essential information that assists in understanding the missing link of lncRNAs to the classical Wnt signaling in CRC.

## 1. Introduction

Colorectal cancer (CRC), accounting for 8% of new cancer cases, is the third most frequent cancer, and one of the leading cause of cancer-related mortalities in the United States [1]. Despite the fact that the CRC incidence rates declined in people aged 50 years or older, CRC incidence rates increased by 22% from 2000 to 2013 in adults younger than 50 years in the United States [2]. Similarly, CRC mortality rates increased by 13% in those less than 50 years old during the same period [2]. The rise of CRC with early age at diagnosis underlines the need for exploration of new avenues for disease understanding, and the development of innovative detection and intervention strategies. 

Aberrant Wnt signaling is the initiating and driving event underlying the vast majority of CRC carcinogenesis [3,4,5]. Although the essential molecules of Wnt signaling have been well characterized, how this pathway is fine-tuned at other layers remains largely unexplored. Non-coding RNA transcripts such as microRNAs have been revealed to restrain or activate Wnt signaling, by controlling the expression of Wnt signaling proteins [6,7,8]. More recently, long non-coding RNAs (lncRNAs), defined as being at least 200 nucleotides in length, show significant association with CRC incidence, the extent of malignancy, and patient prognosis [9,10]. However, the mechanisms underlying lncRNA involvement in CRC lag far behind its discovery pace and remain largely uncharacterized, partially because of the lack of a unified molecular mechanism. 

We reason that because Wnt signaling is an initiating force in CRC carcinogenesis, lncRNAs that participate in this pathway might represent the novel research avenue for mechanisms of understanding the other regulatory layers of CRC. We acknowledge that many excellent reviews have covered each topic of Wnt signaling and lncRNA independently, and refer the readers to this literature for more in-depth information [9,11,12]. In this review, we focus on the knowledge connecting these two topics, and aim to review recent findings linking lncRNAs with essential Wnt signaling. Herein, we briefly introduce the individual topic, summarize the typical examples that connect these two topics, and discuss the clinical application potential of lncRNAs in CRC. We further separated these lncRNAs into several categories: those transcribed from the 8q24 cancer risk region, associated with CRC stem cells, and others. To keep this review concise while informative, we list only the essential findings limited by our own knowledge, and apologize to those whose work was not referenced due to space restrictions.

## 2. Wnt: The Initiating and Driving Force of Colorectal Cancer (CRC)

**Figure 1:**
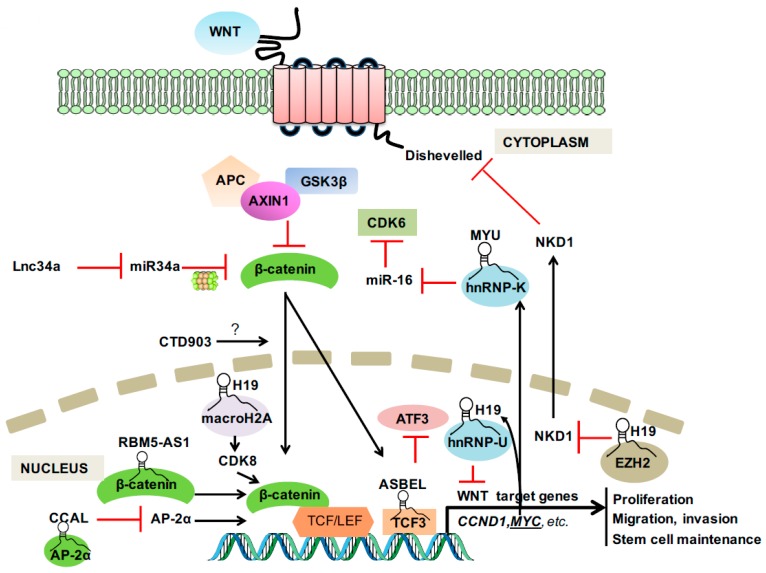
Long non-coding RNA (LncRNA) involvement in canonical WNT signaling. Arrow in black: activate; T in Red: inhibit.

In 1982, the first Wnt family member Int1 (now known as Wnt1) was identified as a mouse proto-oncogene that is activated by the integration of mouse mammary tumor virus [13]. Subsequent research demonstrated that Int1 is a vertebrate homologue of the previously identified fly Wingless (*Wg*) gene, from which the term Wnt originates [14]. As the most well-known and best characterized Wnt signaling pathway, the canonical Wnt signaling that involves β-catenin and members of the lymphocyte-enhancer-binding factor (LEF)/T-cell factor (TCF) family is the fundamental driving force of CRC [15]. In the presence of a Wnt ligand binding to its receptor complex, β-catenin is rescued from ubiquitination and proteasomal breakdown by the destruction complex comprising adenomatous polyposis coli (APC), AXIN1, and GSK3β [16,17]. The stabilized β-catenin translocates to nucleus and forms a complex with LEF/TCF transcription factors to activate the transcription of a wide range of Wnt target genes [18] (Figure 1).

The Cancer Genome Atlas (TCGA) consortium revealed that the Wnt signaling pathway was altered in more than 90% of CRC tumors, with mutational inactivation of the APC tumor suppressor gene, located at chromosome 5q21-q22, in ~80% of cases [19]. As an initiating event in both familial adenomatous polyposis and sporadic CRCs [20,21], the mutational inactivation of APC leads to the accumulation of β-catenin in the nucleus, a hallmark of the canonical Wnt signaling, and the transcriptional activation of Wnt target genes by β-catenin/TCF complex [22,23]. Wnt signaling is essential in maintaining the stem cell niche, and high Wnt activity was reported to accurately define the CRC stem cell population [24]. Experimentally, restoration of APC reverted CRC tumorigenic lesions by re-establishing the normal crypt homeostasis, even in mice harboring oncogenic Kras and mutated p53 [25]. This experimental finding not only reinforces the essential suppressor function of APC in CRC initiation, but also revealed the critical importance of APC-regulated Wnt signaling in CRC progression. As classical downstream targets that respond to Wnt signaling, *CCND1* and *MYC* are established drivers in CRC formation by regulating cell growth, apoptosis, migration, invasion and stem cell maintenance [26,27].

## 3. LncRNAs: The Emerging Dark Matters That Matter

The vast majority of the human genome is transcribed into RNA transcripts, but only a small proportion of these RNA molecules are translated into proteins [28]. The genes that do not code for proteins produce non-coding RNAs (ncRNAs) as the final output. As one type of ncRNA, microRNA (miRNA) received much attention in the last 15 years since the discovery of their cancer involvement [29]. More recently, long ncRNAs (lncRNAs), containing no less than 200 nucleotides, have emerged as important new players in cancer [12,30]. According to their genomic features, these lncRNAs can be further classified into long intergenic ncRNAs (lincRNAs), transcribed ultraconserved regions (T-UCRs), circular RNAs, promoter-associated RNAs, enhancer-associated RNAs, and others [12,31]. In this review, we use the term lncRNA for consistency, although most of the examples in the text are lincRNAs.

A previous study summarized the diverse molecular mechanisms of lncRNAs into four archetypes [32]: (i) lncRNA can serve as a molecular sensor to deliver the signal from the hint of cellular context in a temporal and spatial manner; (ii) lncRNA can serve as decoy to interfere the function of proteins, or that of miRNAs by sponging the miRNAs via sequence complementarity; (iii) lncRNA can guide chromatin-modifying proteins onto target genes, either locally in *cis* or distantly in *trans*, respectively; (iv) lncRNAs can bridge multiple proteins together to modify chromatin or stabilize subcellular structures. It should be noted that these archetypes are not exclusive, and a single lncRNA may have multiple mechanisms.

## 4. LncRNAs in Wnt Signaling and CRC

Since Wnt signaling is an essential pathway in CRC carcinogenesis and progression, it is not surprising that many CRC-associated lncRNAs exert their function via this pathway. The dynamic molecular mechanisms of lncRNAs also render them large diversity in regulating Wnt activity or essential Wnt downstream targets. The 8q24 region represents one of the most frequently amplified cancer-associated regions in CRC, and contains the *MYC* oncogene [33]. Reports in the last several years revealed that the 8q24 region is an oasis for long non-coding RNAs [34,35,36,37,38]. As such, we discuss this unique group of lncRNAs separately. We summarize three lncRNAs in the subsection “CRC stem cells—related lncRNAs” and several lncRNAs that do not share common features in the subsection “Others” (Table 1).

### 4.1. LncRNAs from 8q24 Region

**Figure 2:**
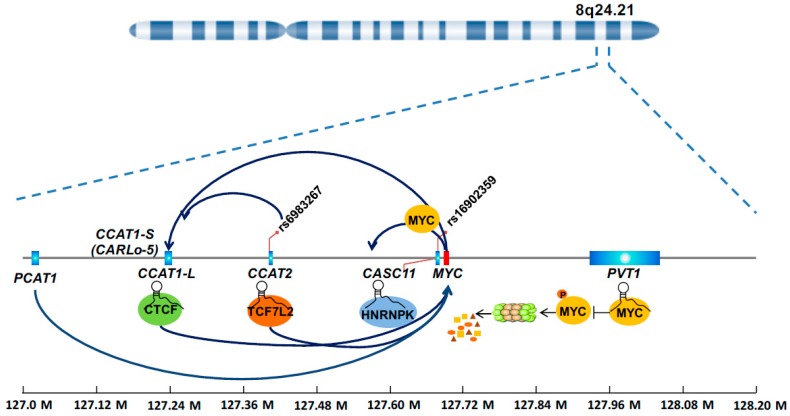
8q24 lncRNAs in MYC regulation.

The chromosome 8q24 is frequently amplified in human cancer. Particularly, the 8q24.21 genomic region that spans almost 2 Mb but represents a desert for protein coding genes and attracts much attention for several reasons. (i) This region contains the *MYC* gene, which is a classical Wnt signaling target and an essential oncogene [27,39]. (ii) Genome-wide association studies consistently suggested that multiple single nucleotide polymorphisms (SNPs) in such region are associated with CRC risk [40,41,42]. (iii) DNA elements in this region have various enhancer activities that are affected by SNPs [43,44], and more recently this region was proposed as a typical example of a super-enhancer [45]. These observations, originating from a different angle but pointing to the same genomic locus, indicate that important unidentified molecular culprits reside in such region. Indeed, the last decade witnessed an explosion of discoveries of lncRNAs in the 8q24.21 region, most of which show relevance with multiple types of cancers including CRC. Not surprisingly, a large proportion of these lncRNAs were found to regulate *MYC*, a protein-coding gene in the region, in one way or another (Figure 2). We can envision that strong enhancer activity in this region promotes transcription of lncRNAs because of the presence of abundant transcriptional factors and mediator proteins. These lncRNAs in turn increase the enhancer activity by forming the chromatin loop or bridging the protein partners, thus creating a positive feedback mechanism in controlling *MYC* expression. Because of their sensitivity in responding to oncogenic signals, the lncRNAs themselves are often found to be prognosis factors in predicting the outcome of CRC patients.

#### 4.1.1. CCAT1

The *CCAT1* gene is located 515 kb upstream of the *MYC* oncogene, encodes a short isoform *CCAT1-S* and a long isoform *CCAT1-L*. *CCAT1-S*, also known as *CARLo-5*, is upregulated in all disease stages, including pre-malignant conditions, during CRC transformation [37]. A meta-analysis suggests significant association of increased *CCAT1* expression in tumor samples with poor survival of cancer patients [46]. The expression of *CCAT1-S* is significantly correlated with the allele status of the SNP rs6983267, located telomeric of *CCAT1-S* [37]. In addition, the genomic region containing rs6983267 forms a chromatin loop with the promoter of *CCAT1-S* gene, suggesting a long-range interaction of rs6983267-containing region with *CCAT1-S* promoter in regulating its expression [37]. Experimentally, knockdown of *CCAT1-S* decreased CRC cell growth in vitro and in vivo [37]. Similar to its shorter isoform, *CCAT1-L* is highly expressed in CRC tumors, and promotes CRC growth in xenograft mouse models [47]. *CCAT1-L* enhances *MYC* transcription, as elegantly demonstrated by genome engineered cell lines that overexpressed *CCAT1-L* at its own gene loci [47]. Chromosome conformation capture assay suggests that CCAT1-L facilitates the formation of a long-range physical interaction loop between the *MYC* enhancer and its promoter [47]. Molecular mechanism study revealed an interaction of *CCAT1-L* with CTCF, an essential protein regulating 3D structure of the chromatin [47]. The *CCAT1* gene is also transcriptionally regulated by MYC [34]. These results reveal a complex molecular interaction connecting SNP, enhancer, lncRNA, and protein in controlling *MYC* expression and CRC growth.

#### 4.1.2. CCAT2

The *CCAT2* gene was identified by the Calin laboratory based on several previous observations [38]. First, this high degree of conservation of this genomic region among mammals suggests the functional importance of this locus and associated transcripts [48]. Second, the rs6983267 SNP in this region is one of the most consistently reported, predisposing SNPs in prostate cancer and CRC [41,49,50]. Third, this region, 335 kb centromeric from the *MYC* oncogene possesses strong enhancer activity that is influenced by the rs6983267 SNP variants [43,44]. After cloning and characterization of the gene, *CCAT2* was found to express at higher levels in microsatellite-stable CRC tumors that exhibit chromosomal instability (CIN), than in microsatellite-instable tumors or normal mucosae that lack the CIN feature [38]. This led to the discovery of *CCAT2* initiation of CIN via cell model systems. Experimental data suggest that *CCAT2* not only exerts *cis* regulatory effects on the nearby gene *MYC*, but also interacts with TCF7L2 protein to exert *trans* regulatory effects on Wnt signaling [38]. Together with the DNA element with enhancer activity influenced by the SNP, the CCAT2 RNA contributes to a unique Wnt signaling regulatory network. This DNA-RNA regulatory network may be essential in CRC, as deletion of this genomic region, which results in loss of both DNA elements and RNA transcripts, reduces the number of the intestine polyps in Apc^Min/+^ mice [51]. Recently, this non-coding RNA was reported to exert allele-specific effects on cancer metabolism by interaction with the splicing protein CFIm and ensuring alternative splicing of glutaminase [52]. In addition, multiple meta-analysis studies proved the prognostic value of *CCAT2* in predicting cancer patient survival [53,54,55,56].

#### 4.1.3. CASC11

*CASC11*, also known as *CARLo-7*, is located ~2.1 kb upstream of the *MYC* gene. The *CASC11* gene encompasses the lymphoma predisposition SNP rs16902359. Similar to the above CCAT transcripts, *CASC11* is overexpressed in CRC tumors, and high *CASC11* correlates with large primary tumors and metastasis to lymph nodes [35]. Ectopic expression of *CASC11* promotes CRC growth and metastasis in vitro and in vivo [35]. Mechanism study showed that CASC11 interacts with and increase the stability of heterogeneous ribonucleoprotein K (hnRNP-K), which protects β-catenin from degradation by the destruction complex, and consequently activates WNT/β-catenin signaling [35]. Forming a feedback mechanism, MYC protein binds to the promoter of the *CASC11* gene to activate its transcription [35].

#### 4.1.4. PVT1

Different from most of the non-coding genes upstream of *MYC*, *PVT1* is located 100–500 kb downstream of the *MYC* gene. In addition, different from *CCAT1* and *CCAT2* that regulate *MYC* transcription, PVT1 controls MYC protein levels by protecting the MYC protein from degradation [36]. Specifically, the physical interaction between PVT1 RNA and MYC protein interferes with its phosphorylation at threonine 58, which is essential in leading to MYC protein degradation [36]. As revealed by in vivo chromosome engineering, PVT1 is indispensable for MYC-induced cancer promoting effect [36]. The copy number of *PVT1* and *MYC* gene was co-increased in nearly all CRC cases with *MYC* gene amplification [36]. Depletion of *PVT1* reduced the tumorigenic capacity of HCT116, a *MYC*-driven CRC cell line [36]. In addition, multiple studies reported significant association between *PVT1* expression and CRC malignancies, and pointed to PVT1 as a potential diagnostic and prognostic marker in CRC [36,57]. Interestingly, the *PVT1* gene locus also harbors a cluster of six annotated microRNA genes (namely, *miR-1204*, *miR-1205*, *miR-1206*, *miR-1207-5p*, *miR-1207-3p*, and *miR-1208*), of which the function remains to be clarified [58]. 

#### 4.1.5. PCAT1

The *PCAT1* gene is located ~725 kb upstream of the *MYC* oncogene. Besides the involvement of *PCAT1* in prostate cancer [59,60], *PCAT1* was also found to be overexpressed in CRC tumors [61]. Experimental data showed that downregulation of *PCAT1* inhibits CRC growth in vitro and in vivo, partially via its regulatory effect on MYC [62]. High levels of PCAT1 expression in primary CRC tumors were significantly associated with distal metastasis of CRC. Moreover, multivariable analysis revealed that increased *PCAT1* expression was an independent factor for poor prognosis in CRC patients [61].

### 4.2. CRC Stem Cell—Associated LncRNAs

Wnt signaling is a determining factor in CRC stem cell maintenance [24,63]. It controls not only essential stem cell genes such as *LGR5*, but also regulates asymmetric division of CRC stem cells [64,65]. The control of CRC stem cells by Wnt signaling offers growth and selection advantages, which may underlie the resistance of CRC tumors to chemotherapeutic drugs [66,67]. Several lncRNAs have been reported to control the fate of the CRC stem cells. 

#### 4.2.1. Lnc34a

Loss of miR-34a is commonly seen in many types of cancer [68,69]. Several reports showed that miR-34a directly targets genes involved in Wnt signaling, including Wnt ligands and the essential β-catenin/TCF7L2 components, resulting in suppression of Wnt activity [7,70,71]. Recently, miR-34a was revealed to be directly involved in controlling CRC stem cell asymmetric division by forming a feedforwarded loop targeting Numb and Notch [72]. In an effort to trace the mechanism of reduced miR-34a expression in CRC cells, a new lncRNA termed lnc34a, transcribed in the opposite orientation from the miR-34a, was identified as a key regulator of miR-34a [73]. Lnc34a interacts with several epigenetic regulators, namely, Dnmt3a, HDAC1, and PHB2, to silence the transcription of miR-34a independent of the p53 protein [73]. The functional importance of lnc34a was demonstrated by its enrichment in CRC stem cells, and its ability to initiate asymmetric division by suppressing miR-34a [73]. Interestingly, lnc34a is distributed unevenly during cell division, and represses the transcription of miR-34a in only one daughter cell [73]. Mouse studies proved the function of Lnc34a in regulating self-renewal of cancer stem cell and CRC growth [73]. Concordantly, lnc34a expression was found to be upregulated in clinical samples of late-stage CRCs [73]. This provides an example of lncRNA-miRNA interaction in maintenance of cancer stem cell feature by regulating Wnt and Notch signaling.

#### 4.2.2. RBM5-AS1

The lncRNA RBM5-AS1 was found to be enriched during sphere formation of colon cancer initialing cells [74]. Silencing of RBM5-AS1 impaired Wnt signaling, while overexpression enhances Wnt signaling in CRC cells [74]. The RBM5-AS1 activity on Wnt signaling is critical for enabling the function of CRC stem cells, as loss of RBM5-AS1 impaired the spheroid formation in multiple CRC cell lines [74]. Mechanism study revealed that RBM5-AS1 physically interacts with β-catenin, and promotes the interaction of β-catenin with the TCF7L2 complex [74]. As a result, Wnt target genes such as *SGK1*, *YAP1* and *MYC* are transcriptionally activated by RBM5-AS1 [74].

#### 4.2.3. WiNTRLINC1

ASCL2 is an essential transcription factor in controlling the stemness of intestinal cells in response to Wnt signaling [65]. Using ChIP-seq with antibodies against RNA polymerase II, *WiNTRLINC1* (WNT-regulated lincRNA 1), located ~60 kb away from the *ASCL2* gene, was identified as one of the direct β-catenin/TCF7L2 targets in CRC [75]. WiNTRLINC1 physically interacts with β-catenin/TCF7L2 to facilitate the looping of regulatory elements, and consequently activate the transcription of the *ASCL2* gene [75]. The regulatory network of Wnt-WiNTRLINC1-ASCL2-stemness is further enhanced with the transcriptional activation of WiNTRLINC1 by ASCL2 [75]. The expression levels of WiNTRLINC1 and ASCL2 were both increased in clinical CRC tumors, and high levels of WiNTRLINC1 were correlated with increased metastatic potential and worse prognosis of CRC patients [75].

### 4.3. Others

#### 4.3.1. H19

*H19* is one of the first imprinted non-coding genes discovered in 1990s [76]. *H19* is exclusively transcribed from the maternally inherited allele and participates as a key factor in embryonic development [77,78]. Various reports have suggested the involvement of *H19* in human cancer [77,79,80,81]. *H19* exerts its function by interaction with EZH2 [82], sponging microRNAs such as let7 and miR-106a [83,84], or production of miR-675 as a primary transcript [85]. Studies from our own work using TCGA CRC data identified *H19* as a top candidate in association with worse CRC survival [86]. Knockdown of *H19* caused a dramatic reduction of CRC cell proliferation and migration [86]. An unbiased approach with microarray analysis revealed not only the known mechanisms of *H19* regulation on let7 and MYC, but also a novel mechanism where *H19* regulates β-catenin activity via modulating CDK8 expression [87], which is probably a consequence of *H19* interaction with the repressive histone variant macroH2A [86,88]. This study, together with other findings that *H19* regulates Wnt signaling by interaction with hnRNP resulting in suppressed expression of *Wnt* genes in liver cells [89], and by interaction with EZH2, leading to Wnt activation through NKD1 repression in bladder cancer [82], provides a vivid example of diverse mechanisms by a single lncRNA. *H19* itself was transcriptionally controlled by the MYC protein, thus forming a connecting loop of Wnt-MYC-H19-Wnt [90]. Multiple studies, including our own work, suggest that *H19* is an independent prognostic marker for CRC survival [86]. Combined analysis of *H19* with its molecular targets significantly improved the prediction power to a level comparable to stage, validating the clinical significance of CDK8-β-catenin regulation by *H19* in CRC [86]. 

#### 4.3.2. CCAL

LncRNA expression profiling of normal, adenoma, and carcinoma tissues identified *CCAL* as a crucial regulator of CRC carcinogenesis [66]. In addition, high CCAL levels in the CRC tumor correspond to short overall survival and poor response to adjuvant chemotherapy [66]. The interaction of CCAL with AP-2α protein promotes the degradation of AP-2α, a negative regulator of β-catenin/TCF7L2 interaction in CRC, and thus indirectly activates Wnt signaling [66]. As a consequence, the multidrug resistance (*MDR1*) gene, which is a Wnt target that encodes the P-glycoprotein 1, is activated [66]. The effect on MDR1 offers a possible explanation on the observed association between *CCAL* expression and therapeutic outcome in patients with CRC.

#### 4.3.3. CTD903

*CTD903* is transcribed from the region of chromosome 14q11.2. Ectopic expression of *CTD903* inhibits cell proliferation and cell motility of CRC cells [91]. Cell line model showed that downregulation of *CTD903* results in the activation of Wnt/β-catenin signaling, and consequently leads to epithelial mesenchymal transition (EMT), as evidenced by the increase of Twist, Snail, and Vimentin, and reduction of the epithelial marker ZO-1 [91]. This effect of *CTD903* on Wnt and EMT provides a possible explanation on the observed suppression of CRC cell invasion by CTD903 [91]. Concordantly, *CTD903* predicts the favorable prognosis of CRC patients [91]. The exact molecular mechanism by which *CTD903* inhibits Wnt signaling remains to be characterized.

#### 4.3.4. ASBEL

In an effort to identify Wnt-regulated lncRNAs in CRC, *ASBEL* (a lncRNA also known as BTG3-AS1) was revealed to be a direct target of β-catenin by RNA-seq and ChIP-seq analysis [92]. Knockdown of *ASBEL* retarded tumor growth in a xenograft mouse model of CRC [92]. Mechanism studies showed that *ASBEL* forms a complex with TCF3, a transcription factor that is transcriptionally activated by β-catenin, to cooperatively suppress *ATF3* gene transcription [92]. This β-catenin-ASBEL-TCF3-ATF3 signaling was demonstrated to be required for CRC proliferation [92]. Together, these data suggest an important role of lncRNA in promoting CRC formation by mediating and executing Wnt activity.

#### 4.3.5. MYU

*MYU* (c-Myc-upregulated lncRNA), originating from opposite strand of the *VPS9D1* gene, is transcriptionally activated by the Wnt target MYC [93]. Downregulation of MYU inhibits the proliferation of MYC-overexpressing cells in vitro and retards xenograft CRC tumor growth in vivo [93]. MYU interacts with the protein hnRNP-K, which binds to the 3′ untranslated region (UTR) of the CDK6 mRNA to prevent the degradation of *CDK6* by miR-16 [93]. Consequently, MYU stabilizes CDK6 and controls the cell transition from G1 to S phase [93]. The mechanism of MYC-MYU-hnRNP-K-CDK6 provides another layer of complexity connecting Wnt signaling and CRC growth. 

## 5. Potential Clinical Application

Once dark genome matter, lncRNA emerged as an important layer of regulation of the essential signaling in CRC [94]. Many of the Wnt-associated lncRNAs exhibit significant association with clinical parameters of CRC. Being potential candidates, lncRNAs have several advantages as cancer biomarkers. First, tissue-specific patterns of lncRNA expression suggests that lncRNAs may be over-represented in specific types of tumor, as exemplified by the successful development of PCA3 as cancer biomarker to help determine the necessity for repeated prostate biopsies [95]. Second, lncRNAs are sensitive signaling molecules in response to tissue-specific and context-specific stimuli [32]. Genentech researchers identified *CCAT1* as a reliable marker for predicting the response to JQ1, a chemical inhibitor of bromodomain containing 4 (BRD4), suggesting the biomarker potential of *CCAT1* in stratifying patients for clinical trials [96]. Third, the fact that some lncRNA genes contain predisposition SNPs offers a unique opportunity for using both DNA and RNA information as biomarkers [31]. As an example, the combined detection of rs6983267 allele status and *CCAT2* levels might strengthen the disease connection. Indeed, a large-scale study showed that the preventative effect of aspirin in CRC was affected by the rs6983267 status [97]. We hypothesize that the *CCAT2* transcript might work together with its DNA counterpart to regulate nearby genes, and influence the effect of aspirin in preventing CRC. Last, a combination of lncRNA profiling with other RNA or protein expression might improve the biomarker sensitivity or specificity. The finding that levels of *H19* and its targets together have a prediction power for CRC prognosis similar to tumor stage supports this possibility, and highlights the importance of mechanism understanding in translational applications [86].

Because of the diverse mechanisms of lncRNAs in fine-tuning Wnt signaling, lncRNAs can be potential therapeutic targets. For instance, the multifaceted function of *H19* (sponging let7 [84], regulating methylation at a genome wide level [98], targeting CDK8-β-catenin signaling [86], and regulating the cell cycle [86]) in CRC makes it possible to tackle multiple oncogenic mechanisms with a single hit on this lncRNA. The unique mechanism of *PVT1* in controlling MYC protein stability can also be explored for therapeutic modulation of MYC protein, which is hard to inhibit by small molecule chemicals [99]. Similarly, finding out how the transcription of lnc34a is controlled might offer novel insights and strategies to target CRC cancer stem cells. 

There are tremendous challenges ahead for the application of the lncRNA in clinical settings: their expression levels are usually low; some intron-derived or nuclear-localized lncRNAs are unstable with a half-life less than 2 h (http://stability.matticklab.com) [100]; their tissue-specific expression pattern may render complexity in the interpretation of profiling by mixed cell population; targeting lncRNAs that reside in the nucleus is still facing many technically difficulties and uncertainties [101]. However, with the technical advances in sequencing and in situ hybridization, it is possible to obtain precise tissue-specificity information and spatial expression patterns. Similarly, with the technical advance on siRNA, antisense oligonucleotides (ASOs), and delivery systems, it is possible to overcome technical difficulties and safety concerns. For instance, most ASOs are taken up by the clearance organs such as liver, and thus, targeting CRC represents unmet challenges [102]. The conjugation of colon specific ligands, similar as the improved distribution seen in the hepatocytes by GalNAc ligand [103], might be one strategy for enhancing the targeting potency of siRNA or ASOs for CRC. Understanding the mechanism such as the effect of protein interactions on cellular uptake of modified ASOs is critical in optimizing the delivery of oligonucleotides [104].

## 6. Conclusions

As an essential signaling in CRC initiation and progression, Wnt signaling is involved in all key aspects of cancer biology related to tumor growth, metastasis, and therapeutic response. As such, any layer of regulation of this signaling has significance in determining the cancer cell fate, and the outcome of CRC patients. The emerging concept of lncRNAs as important players in CRC by regulating Wnt singling deserves the attention of both academia and the biotechnological industry. Currently, the mechanism of understanding lncRNAs in CRC is still in its early infancy, and this represents one of the bottlenecks in bringing them to clinical applications. We propose that the mechanism of elucidation should be facilitated by unbiased and high-throughput experimental analysis including gene expression microarray analysis, RNA sequencing, methylation profiling, protein interaction identification, genomic occupation by lncRNAs, and others. In addition, large-scale database analysis will reveal the clinical significance of lncRNA, and offer hints on the molecular mechanisms. Finally, more effort should be put into the study of the secondary and higher-order structure of lncRNAs, which is critical for detailed understanding of lncRNA function at a molecular level. We expect that the advances on mechanism of understanding lncRNAs in Wnt signaling might bring novel candidates as biomarkers and therapeutics for CRC. We predict that microdissection and single-cell sequencing might represent a trend in fully extracting gene expression information, and foresee that breakthroughs in using oligonucleotides as drugs in the near future will also help achieve the full potential of lncRNA targeting as cancer therapy.

## Figures and Tables

**Table 1:** LncRNAs involved in Wnt signaling and CRC.

LncRNA	Loci	Length	Indentification Method	Mechanism Related to Wnt	Clinical Relevance
**8q24 region**					
CCAT1-S (CARLo-5)	8q24.21	2628 nt	RACE qRT-PCR	Transcription of CCAT1-S is influenced by the allele status of the single nucleotide polymorphisms (SNP) rs6983267 via a long-range interaction of CCAT1-S promoter with rs6938267-containing region.	Promotes CRC growth and invasion; Increased expression correlates with poor prognosis.
CCAT1-L	8q24.21	5200 nt	RNA-seq qRT-PCR Northern blot RACE	Interacts with CTCF to faciliate chromatin looping connecting *MYC* enhancer and promoter, resulting in *MYC* transcription.	
CCAT2	8q24.21	340 nt	qRT-PCR Northern blot RACE	Interacts with TCF7L2 to promote *MYC* and other Wnt target gene transcription. Spans the SNP rs6983267 alleles that responds differentially to Wnt signaling.	Promotes CRC growth and metastasis; Increased expression correlates with poor prognosis.
CASC11 (CARLo-7)	8q24.21	872 nt	qRT-PCR	Interacts with heterogeneous ribonucleoprotein K (hnRNP-K) to protects β-catenin from degradation, and consequently activates Wnt signaling. MYC binds to the promoter of CASC11 to activate its transcription.	Promotes CRC growth and invasion; Increased expression correlates with CRC size, invasion, and lymph metastasis.
PVT1	8q24.21	1957 nt	RACE Northern blot qRT-PCR	Interacts with MYC protein to prevent MYC phosphorylation and degradation.	Promotes CRC growth. Increased expression correlates with poor prognosis.
PCAT1	8q24.21	1992 nt	qRT-PCR, RNA-seq	Increases MYC expression.	Promotes CRC growth.
**CRC stem cell**					
Lnc34a	1p36.22?	693 nt	qRT-PCR RACE	Interacts with Dnmt3a, HDAC1, and PHB2E to epigenetically silences miR-34a expression, resulting in CRC stem cell asymmetric division.	Enriched in CRC stem cells, and upregulated in late-stage CRCs.
RBM5-AS1	3p21.31	1386 nt	lncRNA array RNA-Seq qRT-PCR	Interacts with β-catenin, and promotes the transcriptional activity of β-catenin/TCF7L2 complex.	
WiNTRLINC1	11p15.5	4117 nt	qRT-PCR Northern blot	Interacts with TCF7L2/β-catenin to form chromatin loop and activate ASCL2 transcription.	Increased expression correlates with metastatic potential and poor prognosis.
**Others**					
H19	11q15.5	6295 nt	RACE cloning Northern blot qRT-PCR	Interacts with macroH2A to derepress transcription of CDK8, which positively regulates β-catenin activity. Interacts with hnRNP U to repress Wnt gene transcription. Interacts with EZH2 to repress NKD1 transcription, resulting in Wnt activation. Antagonizes the inhibition of let7 on MYC, which regulates H19 transcription.	Increased expression correlates with poor prognosis independent of other factors.
CCAL	Chr3	1933 nt	Microarray RACE qRT-PCR	Interacts with and degrades AP-2α, a negative regulator of Wnt activity, resulting in increased MDR1 transcription.	Increased expression correlates with poor prognosis and poor response to adjuvant chemotherapy
CTD903	14q11.2	903 nt	Microarray qRT-PCR	Inhibits Wnt signaling and EMT by unknown mechanisms.	Increased expression correlates with favorable prognosis.
ASBEL	21q21.1	2000 nt	qRT-PCR Northern blot	Interacts with TCF3 to repress ATF3 transcription.	
MYU	16q24.3	6310 nt	RNA-seq	Upregulated by MYC. Interacts with hnRNP-K to stabilize CDK6 mRNA.	

* RACE: Rapid amplification of cDNA ends; CTCF: CCCTC-binding factor.
